# Frequency and outcomes of gastrostomy insertion in a longitudinal cohort study of atypical parkinsonism

**DOI:** 10.1111/ene.16258

**Published:** 2024-02-26

**Authors:** Christopher Kobylecki, Viorica Chelban, Yee Yen Goh, Emilia Michou, Riona Fumi, Marte Theilmann Jensen, Rahema Mohammad, Alyssa Costantini, Nirosen Vijiaratnam, Samantha Pavey, Nicola Pavese, P. Nigel Leigh, James B. Rowe, Michele T. Hu, Alistair Church, Huw R. Morris, Henry Houlden

**Affiliations:** ^1^ Division of Neuroscience, Manchester Academic Health Science Centre University of Manchester Manchester UK; ^2^ Department of Neurology, Manchester Centre for Clinical Neurosciences Northern Care Alliance NHS Foundation Trust Salford UK; ^3^ Department of Neuromuscular Diseases, Queen Square Institute of Neurology University College London London UK; ^4^ Neurobiology and Medical Genetics Laboratory “Nicolae Testemitanu” State University of Medicine and Pharmacy Chisinau Republic of Moldova; ^5^ Division of Diabetes, Endocrinology and Gastroenterology University of Manchester Manchester UK; ^6^ Department of Speech and Language Therapy, School of Health Rehabilitation Sciences University of Patras Patras Greece; ^7^ Department of Clinical and Movement Neurosciences, UCL Queen Square Institute of Neurology University College London London UK; ^8^ Multiple System Atrophy Trust London UK; ^9^ Clinical Ageing Research Unit Newcastle University Newcastle UK; ^10^ Department of Neuroscience Brighton and Sussex Medical School Brighton UK; ^11^ Department of Clinical Neurosciences, Cambridge Centre for Parkinson‐Plus, Cambridge University Hospitals NHS Trust University of Cambridge Cambridge UK; ^12^ Division of Neurology, Nuffield Department of Clinical Neurosciences University of Oxford Oxford UK; ^13^ Department of Neurology Royal Gwent Hospital Newport UK

**Keywords:** dysphagia, gastrostomy, multiple system atrophy, parkinsonism, progressive supranuclear palsy

## Abstract

**Background:**

Multiple system atrophy (MSA), progressive supranuclear palsy (PSP) and corticobasal syndrome (CBS) show a high prevalence and rapid progression of dysphagia, which is associated with reduced survival. Despite this, the evidence base for gastrostomy is poor, and the optimal frequency and outcomes of this intervention are not known. We aimed to characterise the prevalence and outcomes of gastrostomy in patients with these three atypical parkinsonian disorders.

**Method:**

We analysed data from the natural history and longitudinal cohorts of the PROSPECT‐M‐UK study with up to 60 months of follow‐up from baseline. Survival post‐gastrostomy was analysed using Kaplan–Meier survival curves.

**Results:**

In a total of 339 patients (mean age at symptom onset 63.3 years, mean symptom duration at baseline 4.6 years), dysphagia was present in >50% across all disease groups at baseline and showed rapid progression during follow‐up. Gastrostomy was recorded as recommended in 44 (13%) and performed in 21 (6.2%; MSA 7, PSP 11, CBS 3) of the total study population. Median survival post‐gastrostomy was 24 months compared with 12 months where gastrostomy was recommended but not done (*p* = 0.008). However, this was not significant when correcting for age and duration of symptoms at the time of procedure or recommendation.

**Conclusions:**

Gastrostomy was performed relatively infrequently in this cohort despite the high prevalence of dysphagia. Survival post‐gastrostomy was longer than previously reported, but further data on other outcomes and clinician and patient perspectives would help to guide use of this intervention in MSA, PSP and CBS.

## INTRODUCTION

The atypical parkinsonian syndromes include multiple system atrophy (MSA), progressive supranuclear palsy (PSP) and corticobasal syndrome (CBS). These syndromes show faster progression and are less responsive to dopaminergic treatment compared with Parkinson's disease (PD). Dysphagia and dysarthria are also more common and severe in these conditions. In patients with pathologically confirmed MSA and PSP, dysphagia occurred sooner after disease onset compared with PD, and was correlated with reduced survival [1]. Dysphagia is associated with increased mortality in both PSP and MSA [2, 3].

There is a limited evidence base for the management of dysphagia in these disorders, including the prevalence and efficacy of gastrostomy insertion. Guidance on the management of dysphagia in MSA, PSP and CBS indicates that gastrostomy insertion can be considered, but the evidence is lacking for outcomes such as improved survival or quality‐of‐life (QoL) [3, 4]. Data on the outcomes of gastrostomy in MSA, PSP and CBS are scarce. A longitudinal study in 59 patients with MSA who had dysphagia identified a mean latency to gastrostomy insertion (done in 42%) of 7 years, with average survival of 1.5 years post‐procedure [5]. In a study of 90 patients with PSP, 20 required non‐oral feeding following aspiration pneumonia, with a mean survival of 2 years following onset of non‐oral feeding [6]. In a retrospective analysis across two tertiary French centres, a total of 15 patients with MSA, 5 with PSP and 3 with CBS underwent gastrostomy over a 7‐year period, with a median survival of 186 days [7]. A recent study of gastrostomy insertion for parkinsonian syndromes in two UK centres over 12 years found a median survival of around 400 days in MSA (*n* = 5) and PSP (*n* = 10) compared with 571 days in PD (*n* = 58) [8].

We aimed to evaluate current practice in the UK by analysing data from a large, longitudinal, multicentre study including patient‐reported outcomes, disease‐specific rating scales and milestones of disease progression. Our objectives were to determine the rate and outcomes of gastrostomy insertion in this cohort, and the progression of dysphagia as measured by disease‐specific rating scales.

## METHODS

We analysed data from participants in the longitudinal and natural history arms of the “Progressive Supranuclear Palsy‐Corticobasal Syndrome‐Multiple System Atrophy” study (PROSPECT‐M‐UK). The methodology of this study has been reported in detail elsewhere [9]. The PROSPECT‐M‐UK study was approved by the London – Queen Square Research Ethics Committee (14/LO/1575) and all participants gave written informed consent to participate.

Participants were classified at baseline into diagnostic categories: MSA, PSP, CBS using consensus clinical diagnostic criteria [10, 11, 12] and an indeterminate atypical parkinsonian group which lacked distinctive clinical diagnostic features at baseline (APS). All participants were assessed at baseline and then at regular intervals (Figure S1). Impact on activities of daily living was assessed across all participants using the Movement Disorder Society‐Unified Parkinson's Rating Scale‐Part II (MDS‐UPDRS‐II) [13] and Schwab and England Activities of Daily Living (SEADL) scale. Motor impairment was assessed in MSA using the Unified Multiple System Atrophy Rating Scale (UMSARS) [14] and in PSP/CBS/APS using the Progressive Supranuclear Palsy Rating Scale (PSPRS) [15]. Cognitive dysfunction was assessed using the Montreal Cognitive Assessment (MoCA) [16].

Baseline impairment of swallowing across the cohort was assessed using question 2.3 (chewing/swallowing) of the MDS‐UPDRS‐II. Questions on dysphagia from the UMSARS Part I and PSPRS historical section were used to assess patient‐reported swallow function in MSA and PSP, CBS and APS, respectively. Latency to gastrostomy insertion was determined by record of study milestones, and survival post‐procedure by the last time seen alive, or date of death, recorded in the study database. As well as assessment of the whole study cohort, we performed a separate analysis of only those who had died or had at least 24 months of study follow‐up to avoid bias to patients with early disease.

Statistical analysis was performed using SPSS Version 28 (IBM). Continuous data were compared using one‐way ANOVA or Kruskal–Wallis test depending on normality of distribution. Categorical data were compared using Chi‐square test. Kaplan–Meier survival curves for gastrostomy outcomes were generated, and survival analysis was controlled for specified covariates using Cox regression analysis. A statistical significance level of *p* < 0.05 was used for all analyses.

## RESULTS

Baseline data were available for 339 patients (PSP 137, MSA 100, CBS 74, APS 28). The study pipeline and numbers at different follow‐up stages are detailed in Figure S1. Patients with MSA were significantly younger at baseline compared with those with other atypical parkinsonian syndromes and had a longer disease duration at recruitment compared with those with PSP (Table 1).

**TABLE 1:** Baseline characteristics of study population.

Characteristic	MSA	PSP	CBS	APS
Patients (*n*)	100	137	74	28
Sex	43 F 57 M	51 F 86 M	47 F 27 M	12 F 16 M
Age at symptom onset (years)	59.2 (9.5)	65.4 (7.3)***	63.3 (7.9)*	65.8 (10.1)***
Duration at baseline (years)	5.1 (2.6)	4.4 (3.1)*	4.5 (2.4)	4.3 (2.3)
UMSARS total score	44.0 (15.2)	–	–	–
PSPRS total score	–	33.3 (13.1)	33.7 (13.0)	20.3 (9.2)
SEADL^a^	60% (40%–80%)^##^	60% (40%–80%)^##^	50% (30%–60%)^###^	80% (60%–90%)
PEG recommended	4	5	0	0
PEG inserted	3	1	0	0
Postural instability	72 (72%)	112 (82%)	41 (55%)	22 (79%)
Using wheelchair outdoors	30 (30%)	32 (23%)	25 (34%)	3 (11%)
Residential or nursing home	0 (0%)	3 (2%)	4 (5%)	2 (7%)
Aspiration pneumonia	4 (4%)	5 (4%)	0 (0%)	0 (0%)
Unintelligible speech	5 (5%)	12 (9%)	4 (5%)	0 (0%)

Abbreviations: APS, atypical parkinsonian syndrome, indeterminate; CBS, corticobasal syndrome; F, female; IQR, interquartile range; M, male; MSA, multiple system atrophy; PEG, percutaneous endoscopic gastrostomy; PSP, progressive supranuclear palsy; PSPRS, Progressive Supranuclear Palsy Rating Scale; SD, standard deviation; SEADL, Schwab and England Activities of Daily Living Scale; UMSARS, Unified Multiple System Atrophy Rating Scale.

^a^ Data are presented as median (IQR). All other continuous data presented as mean (SD).

*p < 0.05, **p < 0.01, ***p < 0.001 vs. MSA; ^##^ *p* < 0.01, ^###^ *p* < 0.001 vs. APS (Kruskal–Wallis test followed by post hoc Dunn's test).

**FIGURE 1:**
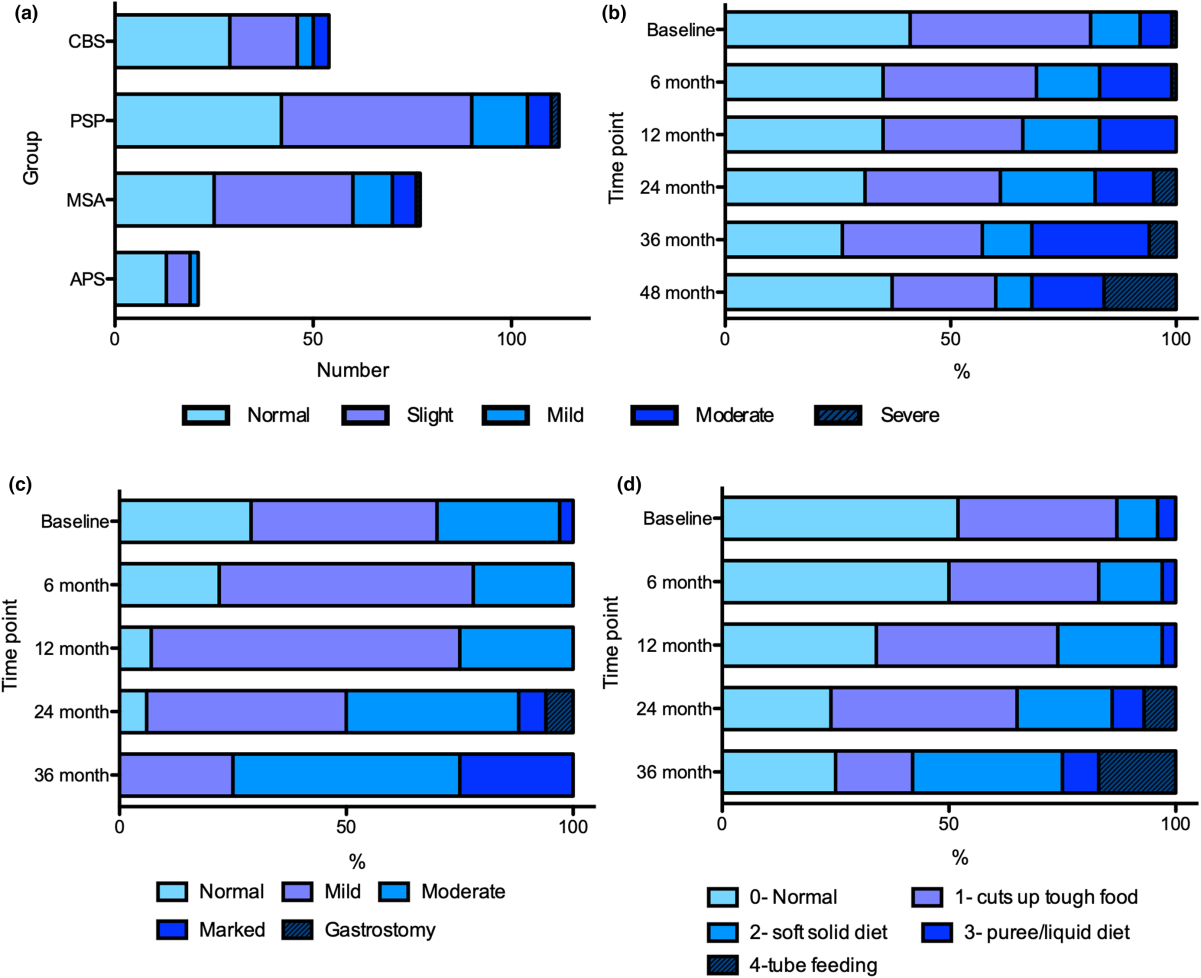
(a) Numbers of patients with progressive supranuclear palsy (PSP), multiple system atrophy (MSA), corticobasal syndrome (CBS) and indeterminate atypical parkinsonism (APS) exhibiting levels of impairment on MDS‐UPDRS‐II question 2.3 (chewing/swallowing) at baseline. (b) Progression of dysphagia across study population as measured by percentage exhibiting levels of impairment on MDS‐UPDRS‐II question 2.3. (c) Progression of dysphagia in MSA patients as measured by UMSARS‐I dysphagia question. (d) Progression of dysphagia in PSP as measured by PSPRS dysphagia question. MDS‐UPDRS, Movement Disorder Society‐Unified Parkinson's Disease Rating Scale.

Swallow dysfunction, as measured across the study population by MDS‐UPDRS‐II question 2.3, was at least mildly impaired at baseline in the majority of participants in all groups (Figure 1a). Few participants had experienced aspiration pneumonia or unintelligible speech at baseline. At baseline, gastrostomy had been recommended in nine patients, and four had undergone this procedure (3 MSA, 1 PSP).

The proportion of the study population showing impairment in swallow increased over the period of study follow‐up (Figure 1b). Whereas at baseline over 70% of patients with MSA exhibited some degree of dysphagia as measured by the UMSARS, <10% of the patients reported normal swallow after 12 months of follow‐up (Figure 1c). Based on the scores from PSPRS, around 50% of patients with PSP had normal swallow at baseline, but by 12 months this had declined to around one‐third and by 24–36 months few remaining participants reported safe swallow (Figure 1d).

Over the follow‐up period, gastrostomy was recommended in 35 more patients (44 in total), and 17 more underwent gastrostomy, giving a total of 21 across the course of the study; their characteristics are summarised in Table 2. Gastrostomy was therefore recommended in 13% of the study population, and performed in 6.2%. When analysing only the 222 participants who had died or had a follow‐up duration of at least 24 months, gastrostomy was recommended in 37 (16.6%) and performed in 18 (8.1%).

**TABLE 2:** Characteristics of patients who underwent gastrostomy insertion.

Characteristic	MSA	PSP	CBS
Patients (*n*)	7	11	3
Sex	6F, 1M	3F, 8M	2F, 1M
Phenotype	MSA‐P 6 MSA‐C 1	PSP‐RS 7 PSP‐P 2 PSP‐F 1 PSP‐CBS 1	
Age at symptom onset (years)	55.0 (5.4)	64.1 (6.3)	61 (15.6)
Duration at baseline (years)	5.8 (1.8)	4.3 (3.6)	5.4 (3.4)
SEADL at baseline	35% (range 10%–50%)	60% (range 20%–90%)	30% (range 30%–90%)
SEADL at gastrostomy	25% (range 10%–50%)	20% (range 10%–40%)	50% (range 10%–90%)^a^
Latency to gastrostomy from onset (years)	6.0 (2.4)	6.7 (3.6)	5.9 (3.1)

*Note*: Data presented as mean (SD) unless otherwise indicated.

Abbreviations: CBS, corticobasal syndrome; F, female; M, male; MSA‐C, multiple system atrophy‐cerebellar; MSA‐P, multiple system atrophy‐parkinsonism; PSP, progressive supranuclear palsy; PSP‐F, PSP‐frontal presentation; PSP‐P, PSP‐parkinsonism; RS, Richardson syndrome; SD, standard deviation; SEADL, Schwab and England Activities of Daily Living Scale.

^a^ Only two of three datapoints available.

A total of seven patients with MSA underwent gastrostomy, predominantly of MSA‐Parkinsonism (MSA‐P) phenotype. Of the 11 PSP patients undergoing gastrostomy, the majority were of PSP‐Richardson syndrome (PSP‐RS) phenotype; three patients with CBS had a gastrostomy inserted. Three patients of those undergoing gastrostomy had eventual pathological confirmation of their diagnosis of PSP.

**FIGURE 2:**
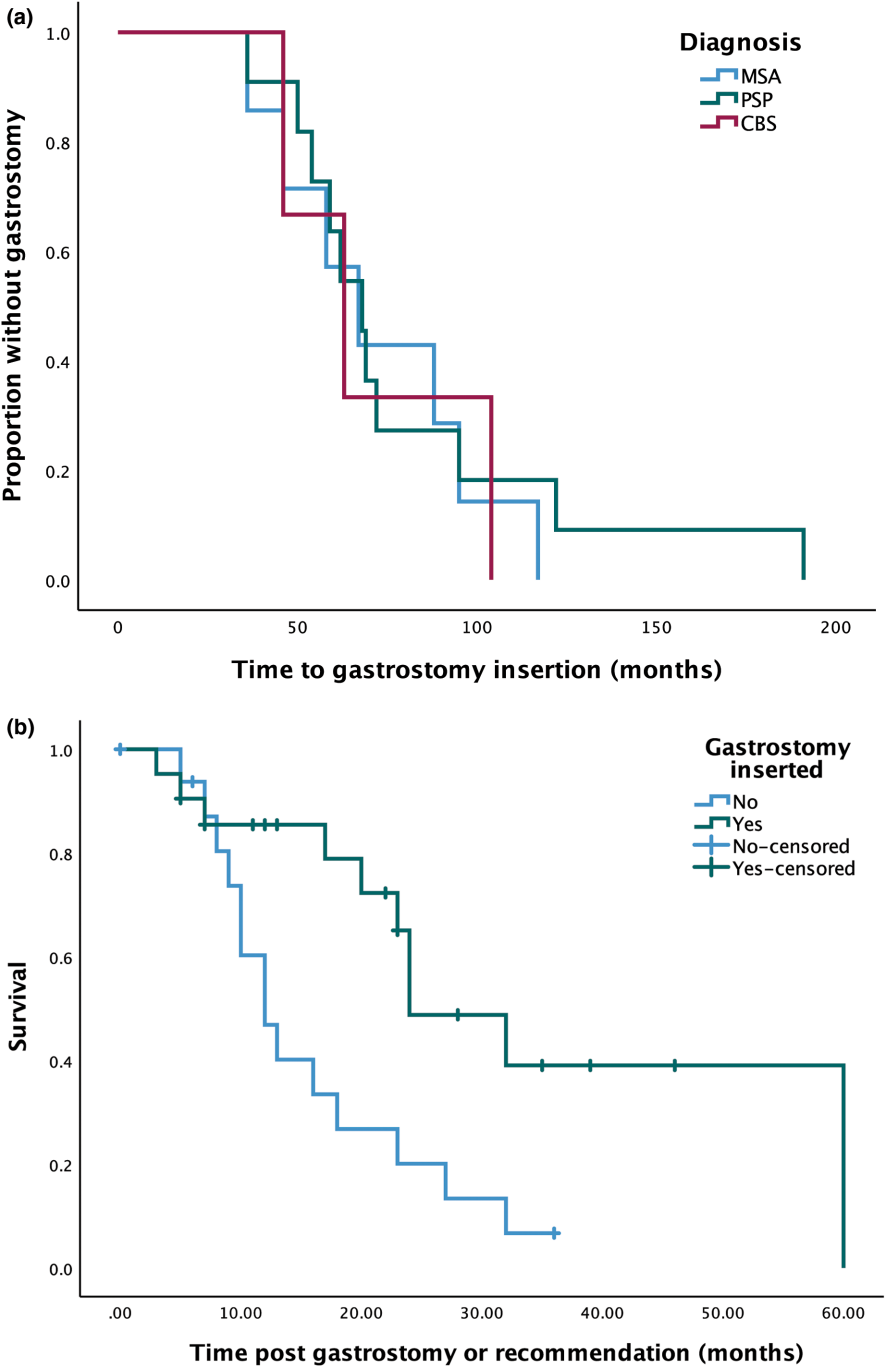
Kaplan–Meier curves showing (a) time to gastrostomy insertion from disease onset in those who underwent the procedure and (b) survival following recommendation for gastrostomy or procedure, stratified by whether gastrostomy was performed. CBS, corticobasal syndrome; MSA, multiple system atrophy; PSP, progressive supranuclear palsy.

Neither MSA nor PSP patients who underwent gastrostomy insertion showed significant differences in age or disease duration at baseline compared with the remainder of their patient group. The median time from symptom onset to gastrostomy was 67 months (95% CI 58.0–76.0; Figure 2a); there were no statistically significant differences in latency between patient groups. There was no significant difference in age, sex or duration of symptoms at baseline between those in whom gastrostomy was inserted post‐recommendation, and those in whom it was not done (Table 3). There was also no statistically significant difference in SEADL scores at the visit preceding gastrostomy insertion between those in whom gastrostomy was and was not performed. MoCA score was significantly lower in those who did not proceed to gastrostomy (19.2 [6.4]) compared with those in whom gastrostomy was performed (23.7 [4.5]; *p* = 0.029).

**TABLE 3:** Comparison between patients in whom gastrostomy was recommended and not performed, and those who underwent gastrostomy insertion.

Parameter	Gastrostomy recommended	Gastrostomy performed
Diagnosis	6 MSA 11 PSP 6 CBS	7 MSA 11 PSP 3 CBS
Sex	11F, 12M	11F, 10M
Age at symptom onset (years)	63.3 (8.1)	60.9 (7.9)
Duration at baseline (years)	4.8 (2.3)	5.3 (3.3)
Total MDS‐UPDRS‐II at baseline	27.3 (10.6)	26.9 (10.3)
SEADL at baseline (%)	50 (range 10–90)	50 (range 10–90)
SEADL at gastrostomy recommendation (%)	20 (range 10–50)	20 (range 10–90)
MoCA at gastrostromy recommendation	19.2 (6.4)	23.7 (4.5)*

Abbreviations: CBS, corticobasal syndrome; MDS‐UPDRS‐II, Movement Disorder Society‐Unified Parkinson's Disease Rating Scale‐Part II; MoCA, Montreal Cognitive Assessment; MSA, multiple system atrophy; PSP, progressive supranuclear palsy; SEADL, Schwab and England Activities of Daily Living Scale.

* *p* < 0.05 compared with gastrostomy recommended.

Of those in whom gastrostomy was performed, 14 (66%) showed evidence of significant postural instability at baseline, whereas 9 (43%) required a wheelchair outdoors. Only one patient at baseline had been admitted to a care home; two exhibited unintelligible speech, and none of the patients had a recorded aspiration pneumonia. At the nearest time point to gastrostomy insertion, 18 patients (86%) required use of a wheelchair outdoors and 5 (24%) had been admitted to either a residential or nursing home. Thirteen patients (62%) had mostly unintelligible speech whereas only three (15%) had an episode of aspiration pneumonia. There was no difference in the frequency of milestones at gastrostomy insertion/recommendation between the two groups. There was a marked decline in median SEADL score, from a median 50% at baseline to 20% at gastrostomy insertion across the cohort as a whole. There were no significant changes in SEADL scores post‐gastrostomy but follow‐up data on this were limited.

The median survival in the gastrostomy group was 24 months (95% CI 14.9–33.1) compared with 12 months (95% CI 8.2–15.8) where gastrostomy was recommended but not performed (log‐rank Chi‐square 7.0; *p =* 0.008; Figure 2b). This difference remained significant when those who had already undergone gastrostomy at baseline were excluded (log‐rank Chi‐square 5.57; *p* = 0.018). Cox regression analysis, controlling for age and symptom duration at time of gastrostomy or recommendation, showed a trend to statistical significance between the two groups (Wald statistic 3.406, *p* = 0.065).

## DISCUSSION

We report data on dysphagia and gastrostomy insertion from a large, longitudinal study of patients with MSA, PSP, CBS and indeterminate parkinsonism. These data arise from a multicentre, observational, prospective‐longitudinal cohort of patients. Survival in those undergoing gastrostomy was longer compared with those where gastrostomy was recommended but not done.

As described in previous studies, swallow dysfunction is common at baseline in all groups and shows rapid progression, with the vast majority of patients having some degree of impairment at 12 months follow‐up. Despite this, recommendation for gastrostomy occurred in only 13% of patients across the follow‐up period of the study, whereas gastrostomy insertion was performed in around 6% of the total population or half of those in whom it was recommended. Even when analysing a more advanced cohort of patients, gastrostomy was still performed in 8% of the cohort. These data provide real‐world information from a large cohort about current practice with regard to nutrition support in patients with MSA, PSP, and CBS.

Our data on swallow dysfunction and progression are in keeping with previously published work highlighting dysphagia as a significant symptom in these conditions [1]. While the MDS‐UPDRS‐II is not a specific scale for the disorders included here, its use across the study groups allows comparison between them. These data, together with disease‐specific scale data, suggest that normal swallow was less common at baseline in MSA compared with PSP; however, the longer symptom duration at baseline in the MSA group may partially explain this observation. Both conditions showed significant deterioration in swallow function within the first year of follow‐up and beyond.

We believe that the information on gastrostomy practice and outcomes provides useful additional data from a large cohort of patients assessed using a standard protocol with a focus on the rates of gastrostomy recommendation or insertion in routine clinical practice. Previous prospective studies largely focused on cohorts of patients selected for dysphagia, so a greater proportion underwent gastrostomy [5, 6]. The data presented here therefore allow an estimate of the current rates of procedure in patients with MSA, PSP and CBS in clinical practice across the UK. In this large, well‐characterised, longitudinal cohort, gastrostomy insertion was performed in up to 8% of cases, and in roughly half of the patients for whom it was recommended. This may reflect the paucity of evidence for intervention, compared with rates of gastrostomy of 14%–60% in motor neuron disease, in which the evidence base for improved survival is greater [17]. However, rather than a registry‐based study, our data were limited to those enrolled in PROSPECT‐M UK, and a more comprehensive registry of patients with MSA, PSP and CBS and their outcomes could help to further resolve this issue.

Direct comparisons with previous studies of gastrostomy outcomes are difficult due to differences in methodology. Marois and colleagues reported a median survival of around 6 months post‐gastrostomy, albeit in a population with a longer median disease duration of 8 years and hence likely to have more advanced disease [7]. The median post‐gastrostomy survival of around 400 days reported in a more recent retrospective study of MSA and PSP is also lower than we report here [8]. However, our data suggest that in selected patients with MSA, PSP and CBS, survival post‐gastrostomy may be longer than previously suggested. The study was not designed to detect changes in quality of life, and future prospective work could usefully focus on this aspect as well as survival.

A novel aspect of our study is the ability to interrogate the study database to identify those in whom gastrostomy had been recommended but not performed. Analysis of survival indicated longer survival in those proceeding to gastrostomy. This is likely to reflect either patient choice, or the identification of patients in whom gastrostomy was subsequently deemed inappropriate due to perceived poor outcome or comorbidities, such as respiratory problems in MSA or dementia in PSP. The available data do not allow these decision‐making processes to be examined in more detail, but do reinforce the importance of being able to provide reliable prognostic information to patients, including on the efficacy of interventions such as gastrostomy. More research on the decision‐making process and optimal timing over gastrostomy would help advance care planning, which is of critical importance in this population, especially in situations where capacity may be lacking [18, 19, 20]. Furthermore, more integration of gastroenterology and nutrition teams into multidisciplinary management of people with atypical parkinsonism, similar to the current situation in motor neuron disease, could enhance planning and management of these aspects.

Our study has some important limitations. First, swallow dysfunction was assessed by patient self‐report rather than objective imaging assessment of dysphagia. However, by analogy with studies in PD we suspect that this would result in underestimation, rather than overestimation, of the prevalence and severity of dysphagia [21]. Second, there was inevitable attrition in longitudinal data due to disease progression and loss to follow‐up, although this still represents a large, longitudinal cohort of patients with atypical parkinsonism. Due to the relatively small numbers undergoing gastrostomy, we were unable to formally analyse changes in health‐related quality of life post‐procedure, which remains an important unmet need in such studies. Third, the study was not able to address the comorbidities present in each group, the rate of complications post‐gastrostomy such as tube blockage or site infections, or the need for tube replacement. Data on the decision‐making process around gastrostomy are also lacking as discussed earlier. Strengths of the study include the longitudinal documentation of disease milestones such as gastrostomy insertion, and recruitment from multiple centres giving a balanced perspective on current practice relating to dysphagia management. In addition, our study provides information on gastrostomy outcome in CBS, for which little evidence hitherto exists in the literature.

## CONCLUSIONS

Swallow dysfunction is common in patients with MSA, PSP and CBS, yet gastrostomy insertion is not commonly recommended and performed. Survival post‐gastrostomy in selected patients may be higher than previously suspected. Further detailed analysis of gastrostomy outcomes, including quality of life, carer burden, and clinician and patient perspectives on this procedure, will be critical to developing understanding of when this intervention is best offered.

## AUTHOR CONTRIBUTIONS

Conceptualisation: C.K., V.C., S.P., E.M., H.H. Data curation: C.K., R.F., M.T.J., R.M., N.V., A.Co. Project administration: C.K., R.F., A.Co. Formal analysis: C.K., V.C., Y.Y.G. Investigation: C.K., V.C., Y.Y.G., M.T.H., J.B.R., A.Ch., N.P., P.N.L., H.R.M., H.H., N.V. Methodology: C.K., V.C., Y.Y.G., H.H., H.R.M. Supervision: H.H., H.R.M. Writing – original draft: C.K., V.C., Y.Y.G. Writing – review and editing: C.K., V.C., Y.Y.G., S.P., E.M., M.T.H., A.Ch., H.R.M., H.H., P.N.L., N.P., J.B.R., H.R.M., R.M., M.T.J., R.F., N.V., A.Co.

## CONFLICT OF INTEREST STATEMENT

Christopher Kobylecki declares no conflict of interest. Viorica Chelban declares no conflict of interest. Yee Yen Goh declares no conflict of interest. Emilia Michou declares no conflict of interest. Riona Fumi declares no conflict of interest. Marte Theilmann Jensen declares no conflict of interest. Rahema Mohammad declares no conflict of interest. Alyssa Costantini declares no conflict of interest. Nirosen Vijiaratnam declares no conflict of interest. Samantha Pavey declares no conflict of interest. Nicola Pavese declares no competing financial interests related to the present article; his contribution to this article reflects entirely and only his own academic expertise on the matter. He has received Advisory Boards honoraria from: Britannia, Bial, Boston Scientific, Benevolent AI, Roche, Abbvie. 4D Pharma; Speaker Honoraria from: Britannia, Abbvie, GE Healthcare, Boston Scientific, the MDS. He has received research grants from the Independent Research Fund Denmark, Danish Parkinson's disease Association, Parkinson's UK, Center of Excellence in Neurodegeneration (CoEN) network award, GE Healthcare Grant, Multiple System Atrophy Trust, Weston Brain Institute, EU Joint Program Neurodegenerative Disease Research (JPND), the MJFF, and the EU Horizon 2020 research and innovation programme. P Nigel Leigh declares no conflict of interest. James B Rowe declares no conflict of interest. Michele T Hu declares no conflict of interest. Alistair Church declares no conflict of interest. Huw R Morris is employed by UCL. In the last 12 months he reports paid consultancy from Roche, Aprinoia, AI Therapeutics and Amylyx; lecture fees/honoraria – BMJ, Kyowa Kirin, Movement Disorders Society. Research Grants from Parkinson's UK, Cure Parkinson's Trust, PSP Association, Medical Research Council, Michael J Fox Foundation. Dr Morris is a co‐applicant on a patent application related to C9ORF72 – Method for diagnosing a neurodegenerative disease (PCT/GB2012/052140). Henry Houlden declares no conflict of interest.

## FUNDING INFORMATION

This study was supported by the MSA Trust and the PSP Association (PROSPECT‐M‐UK). We are grateful to the MSA Coalition, Medical Research Council (MRC UK MR/J004758/1, G0802760, G1001253; MC_UU_00030/14; MR/T033371/1), The Wellcome Trust (equipment and strategic awards WT093205MA and WT104033/Z/14/Z; and 220258) and the Cambridge Centre for Parkinson‐Plus. V.C. received grants from the Multiple System Atrophy Trust/ABN Clinical Research Training Fellowship (Grant F84 ABN 540868), Multiple System Atrophy Trust (PROSPECT‐M‐UK Project), Multiple System Atrophy Coalition (Grant 567540), the Guarantors of Brain (Grant 565908) and the King Baudouin Foundation/Sophia Fund. This research was supported by the National Institute for Health and Care Research (NIHR) Cambridge Biomedical Research Centre (NIHR203312) and the NIHR Oxford Biomedical Research Centre. The views expressed are those of the authors and not necessarily those of the NIHR or the Department of Health and Social Care.

## Supporting information


Figure S1.

## Data Availability

The data that support the findings of this study are available on application to PROSPECT‐M‐UK study. Restrictions apply to the availability of these data, which were used under license for this study.
