# Supplementary figures and images for: Frequency and outcomes of gastrostomy insertion in a longitudinal cohort study of atypical parkinsonism
Source: Eur J Neurol. 2024 Feb 26;31(6):e16258. doi: 10.1111/ene.16258 (PMC11235814; doi:10.1111/ene.16258)

Supplementary figure 1. Study pipeline indicating numbers analysed at different time points.


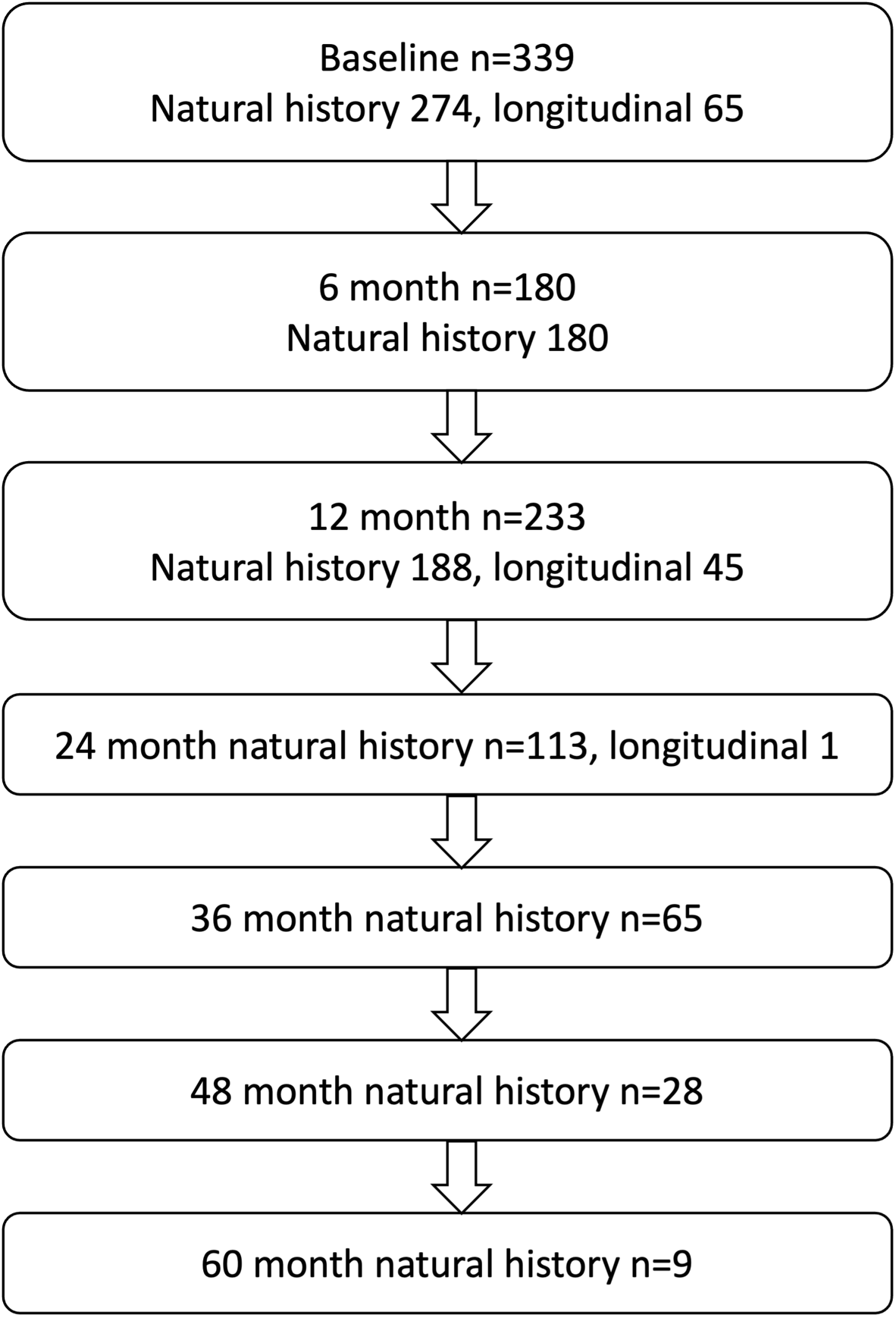

Supplement: Supplementary file 1 — Figure S1. [file ENE-31-e16258-s001.docx]
